# Tibial plateau depression and widening as predictors of meniscal and ligamentous injuries: a systematic review and meta-analysis

**DOI:** 10.1186/s43019-026-00332-6

**Published:** 2026-07-06

**Authors:** Sina Javidmehr, Muhammad Parsa Pashazadeh, Mohammad Reza Ramezanpour, Armin Akbarzadeh, Behnaz Behzadi, Robert F. LaPrade

**Affiliations:** 1 Department of Orthopedic Surgery, Sina Hospital, Tehran University of Medical Sciences, Tehran, Iran; 2 School of Medicine, Tehran University of Medical Sciences, Tehran, Iran; 3 Department of Orthopedic Surgery, School of Medicine, Shiraz University of Medical Sciences, Shiraz, Iran; 4 Department of Radiation Oncology, Mahdiyeh Hospital, School of Medicine, Shahid Beheshti University of Medical Sciences, Tehran, Iran; 5 Twin Cities Orthopedics, Edina, USA; 6 Orthopedic Subspecialty Research Center (OSRC), Tehran University of Medical Sciences, Tehran, Iran

**Keywords:** Tibial plateau fracture, Tibial plateau depression, Tibial plateau widening, Lateral meniscus, Soft-tissue injury

## Abstract

**Background:**

Tibial plateau fractures are complex knee injuries frequently accompanied by meniscal and ligamentous injuries. Although radiography and computed tomography (CT) are routinely used to evaluate fracture patterns, their ability to predict associated soft-tissue injuries has not been well established. This systematic review investigated the diagnostic value of tibial plateau depression and widening as radiographic predictors of meniscal and ligamentous injuries in patients with tibial plateau fractures.

**Methods:**

A systematic review and meta-analysis was conducted in accordance with PRISMA 2020. MEDLINE, Scopus, Embase, and Web of Science were searched up to July 2025. Studies evaluating the association between tibial plateau depression or widening and meniscal or major ligamentous injuries (cruciate and collateral ligaments) in adults were included. Random-effects models were used to pool mean differences (MDs) and diagnostic accuracy estimates.

**Results:**

A total of 17 studies encompassing 1348 patients were included. Patients with lateral meniscus (LM) injuries had significantly greater lateral plateau depression (LPD) than those without (MD 5.03 mm, 95% CI 3.94–6.12). The pooled diagnostic odds ratio (DOR) of LPD for detecting LM injury was 9.80, with a sensitivity of 84%, specificity of 63%, and an area under the sROC curve (AUC) of 0.724. Lateral plateau widening (LPW) was also significantly higher in patients with LM injuries (MD 1.37 mm, 95% CI 0.29–2.45), with a pooled DOR of 7.97, sensitivity of 71%, specificity of 71%, and AUC of 0.758. Associations between LPD/LPW and medial meniscus or cruciate ligament injuries were more heterogeneous, although LPD had consistent relationships with lateral collateral ligament injury and LPW showed a significant pooled association with anterior cruciate ligament (ACL) injury (MD = 3 mm).

**Conclusions:**

LPD was the most consistent radiographic predictor of LM injury, with injured patients demonstrating, on average, 5 mm greater depression, while LPW provided complementary value with a pooled MD of 1.37 mm in patients with LM injury and also showed a significant, though more heterogeneous, association with ACL injury. These routinely available CT- or radiograph-based parameters can help stratify the risk of soft-tissue injury and guide selective use of MRI or arthroscopic inspection with tibial plateau fractures.

**Supplementary Information:**

The online version contains supplementary material available at 10.1186/s43019-026-00332-6.

## Background

Tibial plateau fractures are complex injuries of the knee joint that can result from both low- and high-energy trauma, with the latter usually causing more severe fracture patterns [1, 2]. Such fractures can lead to various soft-tissue injuries, with concomitant ligament and meniscal involvement being among the most concerning [3–7]. These injuries may result from direct high-energy trauma or from indirect mechanisms such as axial compression and torsional loading [5]. Restoring the joint surface, realigning the limb, and reconstructing knee stability are the core objectives of treatment for tibial plateau fractures [8].

Mismanagement of associated meniscal and ligamentous injuries has been linked to substantial long-term morbidity, delayed functional recovery, and an increased risk of post-traumatic osteoarthritis following fracture management [9, 10]. Although open reduction and internal fixation (ORIF) remains the gold standard for addressing complete articular fractures (Schatzker IV–VI) [1], the adjunctive use of arthroscopy offers significant advantages by enabling direct visualization and management of concomitant meniscal and ligamentous injuries, ultimately enhancing clinical outcomes [1, 8, 11]. Consequently, accurate identification and assessment of these injuries in tibial plateau fractures are essential to optimize treatment and prognosis.

Magnetic resonance imaging (MRI) is especially helpful for spotting these soft-tissue injuries [12]. However, the high expense, lengthy wait times, limited availability, and the time-consuming nature of the test itself limit its routine use [13]. In addition, widespread periarticular edema and intra-articular hemorrhage during the acute phase of tibial plateau fractures may impair diagnostic precision [14]. In contrast, computed tomography (CT) is widely used as a standard imaging technique for evaluating tibial plateau fractures [15]; however, its ability to identify concomitant meniscal and ligamentous injuries is limited.

Numerous observational studies have demonstrated that marked tibial plateau displacement, such as articular depression and widening, are associated with an increased risk of meniscal and ligamentous injuries [5, 9, 16–18]. In this context, CT and plain radiographic imaging serve as valuable tools for orthopedic surgeons to accurately evaluate the extent of tibial plateau displacement [18]. Tibial plateau depression and widening represent standardized radiographic parameters in plain radiographic or CT images for evaluating the depression and widening of the plateau. However, to date, no systematic review and meta-analysis has comprehensively evaluated the association between these radiographic parameters and the occurrence of meniscal or ligamentous injuries in patients with tibial plateau fractures. Accordingly, the purpose of this review was to investigate the relationship of tibial plateau depression and widening with the presence of meniscal and major knee ligamentous injuries (cruciate and collateral ligaments), and to determine how accurately these measurements can diagnose these associated soft-tissue lesions.

## Methods

This systematic review and meta-analysis adhered to the Preferred Reporting Items for Systematic Reviews and Meta-Analyses (PRISMA) 2020 guidelines [19] and was prospectively registered in PROSPERO (CRD420251064726).

### Search strategy

A comprehensive search of four electronic databases, including MEDLINE/PubMed, Scopus, Embase, and Web of Science, was conducted to identify studies related to tibial plateau fractures up to July 2025. The search strategy was constructed using Boolean operators in combination with relevant keywords and associated MeSH terms, including “tibial plateau fracture,” “plateau depression,” “plateau widening,” and “soft-tissue injury.” The search strategy underwent critical appraisal by the senior author (SJ) in accordance with the Peer Review of Electronic Search Strategies (PRESS) standards [20]. Supplementary Table S1 provides tailored search strings for each database. Additionally, the reference lists of all included studies were carefully reviewed to identify any further relevant articles that may not have been captured in the database search.

### Eligibility criteria

This review included studies that examined the association between depression or widening of the tibial plateau after fracture and concomitant injuries to the medial and lateral menisci (MM/LM), anterior and posterior cruciate ligaments (ACL/PCL), and medial and lateral collateral ligaments (MCL/LCL). Study eligibility was also assessed according to the PECOS framework [21]. The participants were adults with tibial plateau fractures. The exposures of interest were tibial plateau widening or depression or other radiographic parameters, and the outcomes were concomitant knee meniscal and ligamentous injuries. Eligible study designs encompassed randomized controlled trials (RCTs), nonrandomized trials, prospective and retrospective cohort studies, case–control studies, and case series. Review articles of any type, case reports, letters to the editor, and abstracts were excluded. No restrictions were applied regarding the year of publication, and studies published in languages other than English were excluded.

### Study selection and data extraction

EndNote version 21 was used to manage citations retrieved from the databases [22]. After duplicate records were removed, two independent reviewers (MPP and MRR) screened the titles and abstracts in a blinded manner. The Rayyan platform was employed at this stage to maintain updated records and facilitate the selection process [23]. Articles deemed potentially eligible then underwent full-text assessment by a third reviewer (SJ). Discrepancies in study selection were resolved through discussion and review of the full texts. In addition, relevant articles identified through citation screening were also considered for inclusion.

A standardized spreadsheet, designed according to the objectives of the review and characteristics of the included studies, was developed under the supervision of the senior author (SJ) for data extraction. Data was taken from the eligible publications by two independent reviewers (MPP and MRR) to ensure accuracy and consistency. The senior author (SJ) examined and addressed any inconsistencies. Extracted data included general study characteristics and patient demographics (sample size, sex distribution, age, and body mass index [BMI]). Additionally, information on tibial plateau widening and depression, the imaging modality used for their assessment, associated soft-tissue injuries, and the reference standard employed for diagnosing soft-tissue injuries was collected.

### Risk of bias assessment

We assessed the methodological quality of the included studies using the JBI Checklist for Analytical Cross-Sectional Studies, a tool recommended by the Joanna Briggs Institute (JBI) for systematic reviews [24]. This checklist is recognized as one of the most comprehensive instruments for evaluating the risk of bias in cross-sectional research and has been widely applied in systematic review methodologies. Moreover, the Quality Assessment of Diagnostic Accuracy Studies 2 (QUADAS-2) tool [25] was employed to evaluate the risk of bias in studies included in the diagnostic test accuracy (DTA) analyses.

To ensure a thorough and unbiased assessment, two independent reviewers (AA and BB) applied the checklists to the included studies. Each reviewer independently assessed studies, categorizing the risk of bias within each domain as low, unclear/some concerns, or high. Subsequently, a third reviewer (SJ) reviewed and confirmed the overall quality assessment for each study.

### Data analyses

Quantitative synthesis was only performed when studies reported compatible effect measures (group-wise means and standard deviations [SDs]). Outcomes with insufficient or incompatible data were synthesized narratively.

Meta-analyses were performed to pool between-group differences in lateral plateau depression (LPD) and lateral plateau widening (LPW) and to synthesize diagnostic accuracy estimates of these parameters. To pool the between-group differences in LPD and LPW, mean differences (MDs) were estimated using random-effects models based on the inverse-variance method. When studies reported medians with interquartile ranges (IQRs) or ranges, we converted them to means and SDs using the methods described by Wan et al. and Luo et al. [26, 27]. Subgroup analyses were performed on the basis of the measurement modality (CT versus radiography) and the reference standard used for confirming soft‑tissue injury (arthrotomy/arthroscopy versus MRI). Leave-one-out sensitivity analyses were performed by sequentially omitting each study to evaluate the robustness of the pooled estimates.

A univariate random-effects model was conducted to compute the overall diagnostic odds ratio (DOR) across the DTA studies, which combines sensitivity and specificity independently of disease prevalence. Pooling sensitivities and specificities using a univariate model was avoided, as this approach can be misleading [28]. Instead, the Reitsma bivariate random-effects model was employed to jointly pool sensitivity and specificity [29]. Using the same model, the summary receiver operating characteristic (sROC) curve was generated. For studies containing zero cells, we applied a continuity correction of 0.5 to all cells of the affected 2 × 2 table. The accuracy of the parameters for diagnosing meniscal injuries was assessed using the log DOR and area under the sROC curve (AUC).

Heterogeneity in continuous-outcome meta-analyses was assessed using Cochran’s Q test (*P* < 0.10 indicating statistical heterogeneity) and quantified with the *I*^2^ statistic (≈25%, 50%, and 75% interpreted as low, moderate, and high heterogeneity, respectively). In DTA models, heterogeneity and threshold effects were evaluated via the between-study variance components for sensitivity and specificity and their correlation from the bivariate model; we additionally reported the Holling sample size–adjusted *I*^2^ [30, 31]. Funnel plot asymmetry, Egger’s test, and trim-and-fill method [32] were used to assess publication bias in continuous-outcome analyses, whereas Deeks’ funnel plot asymmetry test (*P* < 0.10 considered significant) was applied to evaluate small-study effects in DTA studies. All data analyses were performed using the “meta” and “mada” packages in R software, version 4.5.1 (R Foundation for Statistical Computing, Vienna, Austria) [33, 34]. A *P* < 0.05 was considered statistically significant.

## Results

**Fig. 1:**
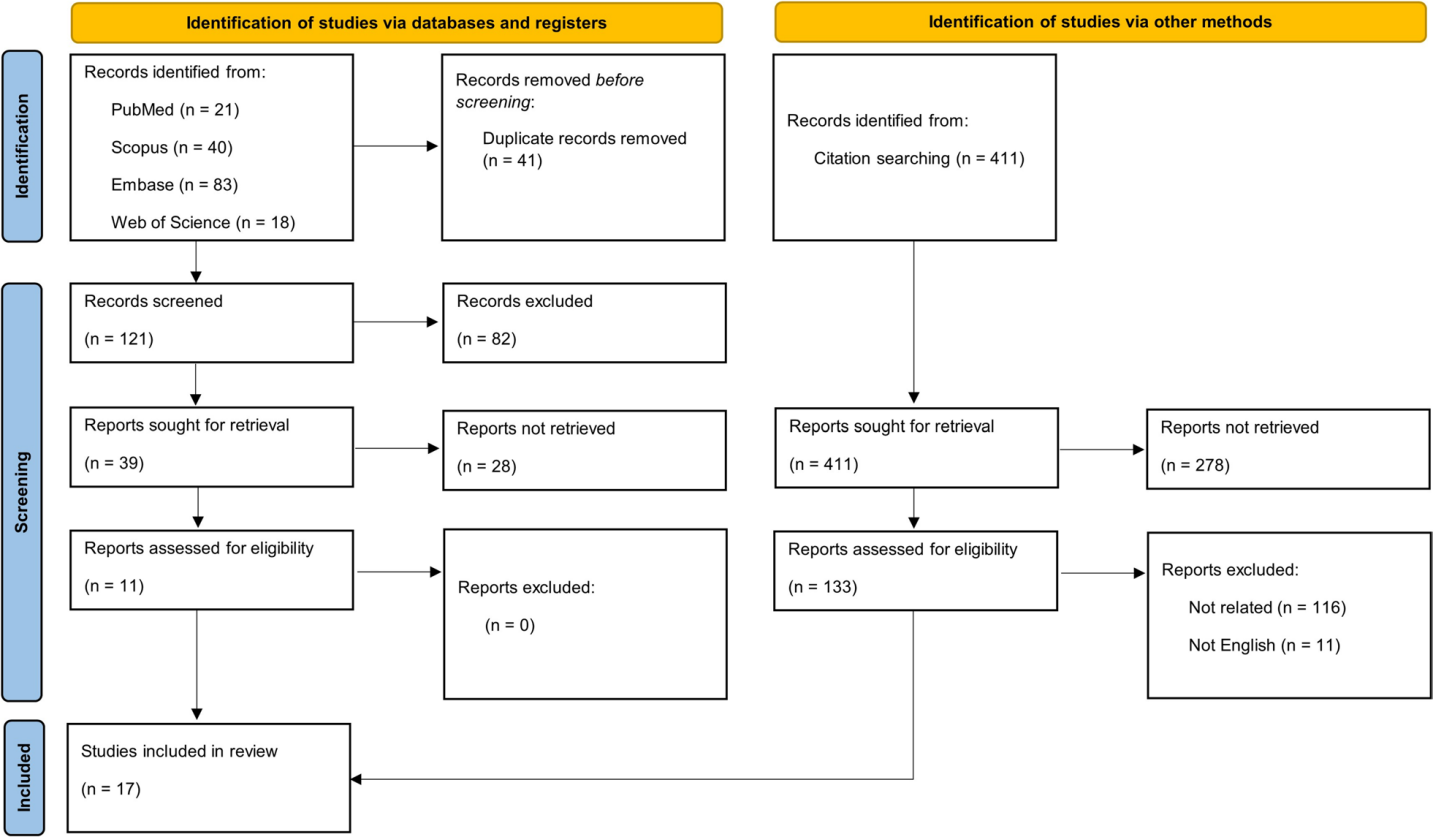
Preferred Reporting Items for Systematic Reviews and Meta-Analyses (PRISMA) flow diagram of study selection

A total of 573 records were identified, including 162 from database searches and 411 through citation screening. After title, abstract, and full‑text review, 17 studies met the inclusion criteria—11 from database searches and 6 from citation screening (Fig. 1)—comprising 1348 patients with tibial plateau fractures. The mean age among the included studies ranged from 40.7 to 52.6 years. Designs included both prospective diagnostic series and retrospective cohorts, corresponding to levels of evidence II–IV. LPW, LPD, and related parameters were measured predominantly using CT (*n* = 13), with some studies using plain radiographs (*n* = 2), both CT and radiographic modalities (*n* = 1), or MRI (*n* = 1). Meniscal and ligamentous injuries were confirmed by MRI (*n* = 6), arthroscopy (*n* = 3), arthrotomy (*n* = 5), or a combination of methods (*n* = 3). Where fracture distribution was reported, 52 cases were Schatzker type I, 736 type II, 105 type III, 159 type IV, 135 type V, and 89 type VI. One study [35] used the AO/OTA system, including 23 patients with isolated lateral plateau (AO 41B) fractures (Tables 1 and 2).

**Table 1:** Demographic characteristics of the included studies

First author (year)	Study type/Level of evidence	Total sample size	Mean age ± SD^a^	Sex (M/F)^b^
Chang (2023) [36]	Prospective diagnostic study/Level II	100	44.6 ± 13.3	77/23
Choi (2018) [37]	Retrospective study/III	53	44.1 ± 10.8	42/11
Durakbasa (2012) [16]	Retrospective comparative study/Level III	20	48.8 ± 37.5	14/6
Gardner (2006) [5]	Retrospective cohort/Level III	62	40.9 ± 13.6	NR^c^
Hassan (2018) [38]	Prospective diagnostic study/Level II	55	40.7 ± 13.3	23/17
Jain (2020) [9]	Retrospective cohort study/Level III	87	45.3 ± 16.5	58/30
Kim (2021) [17]	Retrospective cohort study/Level IV	42	47.2 ± 9.6	26/16
Kolb (2018) [14]	Retrospective case series/Level IV	55	52.6 ± 18.0	18/37
Liu (2023) [18]	Retrospective cohort study/Level III	60	45.3 ± 17.6	35/25
Pu (2022) [39]	Retrospective cohort study/Level III	296	45.0 ± 15.7	174/122
Ringus (2010) [42]	Retrospective cohort study/Level III	85	44.9 ± 17.5	50/35
Salari (2020) [43]	Retrospective cohort study/Level IV	70	45.1 ± 12.9	49/21
Shi (2025) [45]	Retrospective cohort study/Level III	100	42.0 ± 8.8	56/44
Spiro (2013) [44]	Retrospective cohort study/Level IV	54	51.2 ± 18.3	20/34
Tang (2017) [40]	Retrospective cohort study/Level III	132	45.7 ± 13.1	60/72
Tunçez (2023) [35]	Retrospective cohort study/Level IV	23	43.3 ± 8.6	17/6
Wang (2015) [41]	Prospective observational study/Level II	54	48.3 ± 11.2	35/19

^a^*SD* Standard Deviation

^b^*M/F* Male/Female

^c^*NR* Not reported

**Table 2:** Summary of imaging predictors of soft-tissue injuries in tibial plateau fractures

First author (year)	Fracture calcification tool	Specific fracture types reported	Predictor variables	Target injury evaluated	Imaging modality	Final assessment	Main findings
Chang (2023) [36]	Schatzker	I (4), II (33), III (12), IV (22), V (16), VI (15)	dLPD, aLPD, LPW, dMPD, aMPD, MPW, energy level	LM, MM, ACL, PCL	CT	Arthroscopy	High rate of meniscal and ligament injuries; pre-op CT can predict risk and guide arthroscopic inspection
Choi (2018) [37]	Schatzker	I (0), II (21), III (6), V (17), VI (8)	LPD, LPW	LM	CT	MRI	Increased lateral depression and widening correlate with LM tears; MRI advised if these CT signs are present
Durakbasa (2012) [16]	Schatzker	II (20)	LPD, LPW	LM	X-ray	Arthrotomy	Radiographic depression and widening strongly predict lateral meniscus detachment; thresholds have 100% sensitivity and 87% specificity
Gardner (2006) [5]	Schatzker	II (62)	LPD, LPW	LM, MM, ACL, PCL, MCL, LCL, popliteus, posterolateral complex	X-ray	MRI	Soft-tissue injuries rise with increasing displacement; > 5 mm depression/widening should prompt MRI consideration
Hassan (2018) [38]	Schatzker	I (5), II (8), III (7), IV (9), V (6), VI (5)	LPD, LPW	LM, ML, ACL, PCL, MCL, LCL	CT	MRI	LPD correlates with meniscus + ligament tears; LPW mainly correlates with cruciate/collateral tears; multiple injuries increase with both parameters
Jain (2020) [9]	Schatzker	I (1), II (50), IV (19), VI (18)	Depression, widening	Meniscal injuries, especially bucket handle tears	CT	Arthrotomy	Tibial plateau fractures with > 8 mm widening strongly predict meniscal tears; widening was the only significant radiographic indicator of soft-tissue injury
Kim (2021) [17]	Schatzker	II (39), III (3)	Joint depression depth	LM, MM	CT	Arthroscopy	Lateral plateau depression > 6 mm and fracture displacement > 8 mm on CT are independent predictors of ACL and lateral meniscus injuries in tibial plateau fractures
Kolb (2018) [14]	Schatzker	I (4), II (50), III (1)	LPD, LPW	LM, MM, ACL, PCL, LCL, MCL, patellar retinaculum	CT	MRI	MRI and CT correlation showed that greater articular depression significantly increases risk of meniscal and cruciate ligament damage, especially in Schatzker II–IV patterns
Liu (2023) [18]	Schatzker	IV-C only	LPD, LPW	LM	CT	Arthrotomy	CT-measured lateral plateau widening > 7 mm and depression > 6 mm predict combined meniscal and ACL injuries, aiding preoperative assessment
Pu (2022) [39]	Schatzker	II (296)	LPD, LPW	LM	CT	Arthrotomy, arthroscopy	Depression > 10 mm or fragment separation > 8 mm on CT predicts high likelihood of meniscus tears and ligament damage, even without MRI confirmation
Ringus (2010) [42]	Schatzker	I (2), II (21), III (9), IV (1), V (36), VI (16)	LPD	LM	CT	Arthrotomy	Patients with > 10 mm depression had an eight-fold increased risk of lateral meniscus tear; younger than 48 years also had higher tear incidence
Salari (2020) [43]	Schatzker	I (12), II (58)	Articular impaction/displacement (AID, mm), fracture zone (AL, AM, PL, PM)	LM	CT	Arthrotomy	For every 1 mm increase in articular depression (AID), meniscus-tear risk increases by 21%; AID > 4.3 mm gives 100% sensitivity for predicting tears
Shi (2025) [45]	Schatzker	I (15), II (16), III (31), IV (21), V (9), VI (5)	Tibial plateau widening, Fluid accumulation in the joints	ACL	MRI	Arthroscopy, arthrotomy	MRI-based tibial plateau widening and joint effusion volume are accurate for diagnosing ACL injuries, with TPW AUC = 0.826 and joint index AUC = 0.864, correlating with poor postoperative recovery
Spiro (2013) [44]	Schatzker	I (2), II (31), IV (5), V (9), VI (7)	Articular depression	LM, ACL	CT	MRI	Articular depression correlates strongly with both meniscus lateralis and ACL tears; number of soft-tissue injuries rises with degree of depression
Tang (2017) [40]	Schatzker + three-column classification	I (4), II (25), III (20), IV (8), V (52), VI (23)	LPD, LPW, MPD, medial displacement, column involvement	Meniscus, ACL, PCL, popliteal tendon	CT	Arthroscopy	Lateral depression > 11 mm predicts lateral meniscus tear, while medial displacement > 3 mm and anteromedial / posterolateral column involvement predict ACL avulsion
Tunçez (2023) [35]	AO/OTA 41B (lateral plateau)	AO 41B only	Joint depression, LPW	LM, MM, ACL, PCL, MCL, LCL, bucket handle	CT	MRI	
Wang (2015) [41]	Schatzker	I (3), II (27), III (3), IV (14), V (7)	LPD, LPW	Meniscus, ACL, PCL, MCL, LCL	CT and X-ray	MRI, arthrotomy	

### Risk of bias assessment

**Fig. 2:**
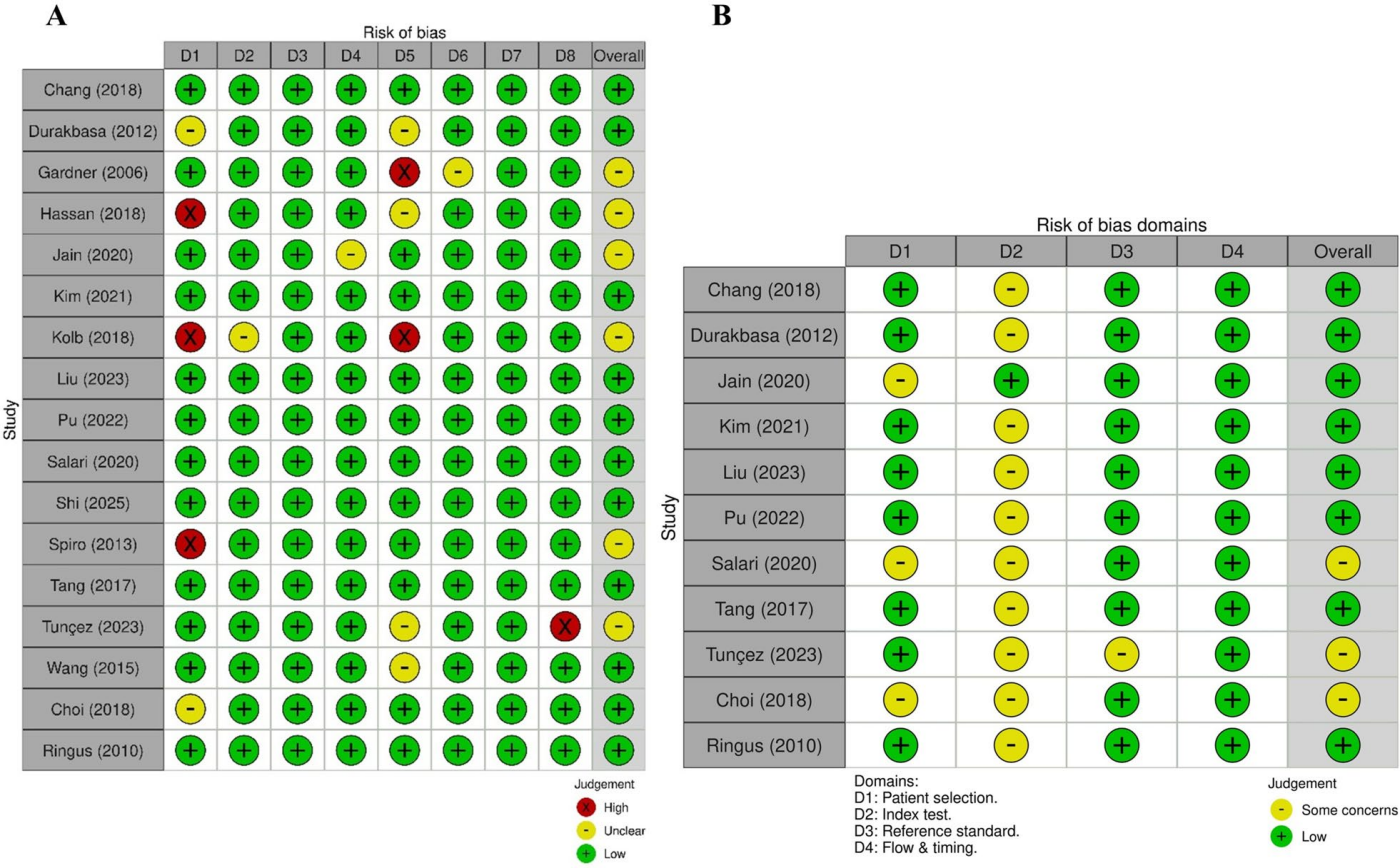
**A** Risk of bias assessment of all included studies using JBI checklist for analytical cross-sectional studies. **B** Risk of bias assessment of diagnostic accuracy studies using QUADAS-2 tool

Based on the JBI checklist, 11 studies were classified as having a low risk of bias, while six studies were deemed to have a moderate risk of bias. Notably, no studies were identified as having a high risk of bias (Fig. 2A). Although some studies exhibited certain limitations in quality assessment, such as inadequate consideration of confounding factors or inadequate reporting of inclusion and exclusion criteria, the overall methodological quality of the included studies was regarded as acceptable. Using the QUADAS-2 tool, 11 studies included in the DTA analysis were evaluated. Three were rated as having an overall judgment of “some concerns” (Fig. 2B). Most studies showed some concerns in the index test domain due to the absence of a prespecified cutoff value, while the remaining domains were generally assessed as having low risk of bias.

### LPD in tibial plateau fractures

A total of 16 studies [5, 9, 14, 16–18, 35–44] measured the LPD as a radiological parameter for predicting soft-tissue injuries, including LM, MM, ACL, PCL, MCL, and LCL.

#### Lateral meniscus

**Fig. 3:**
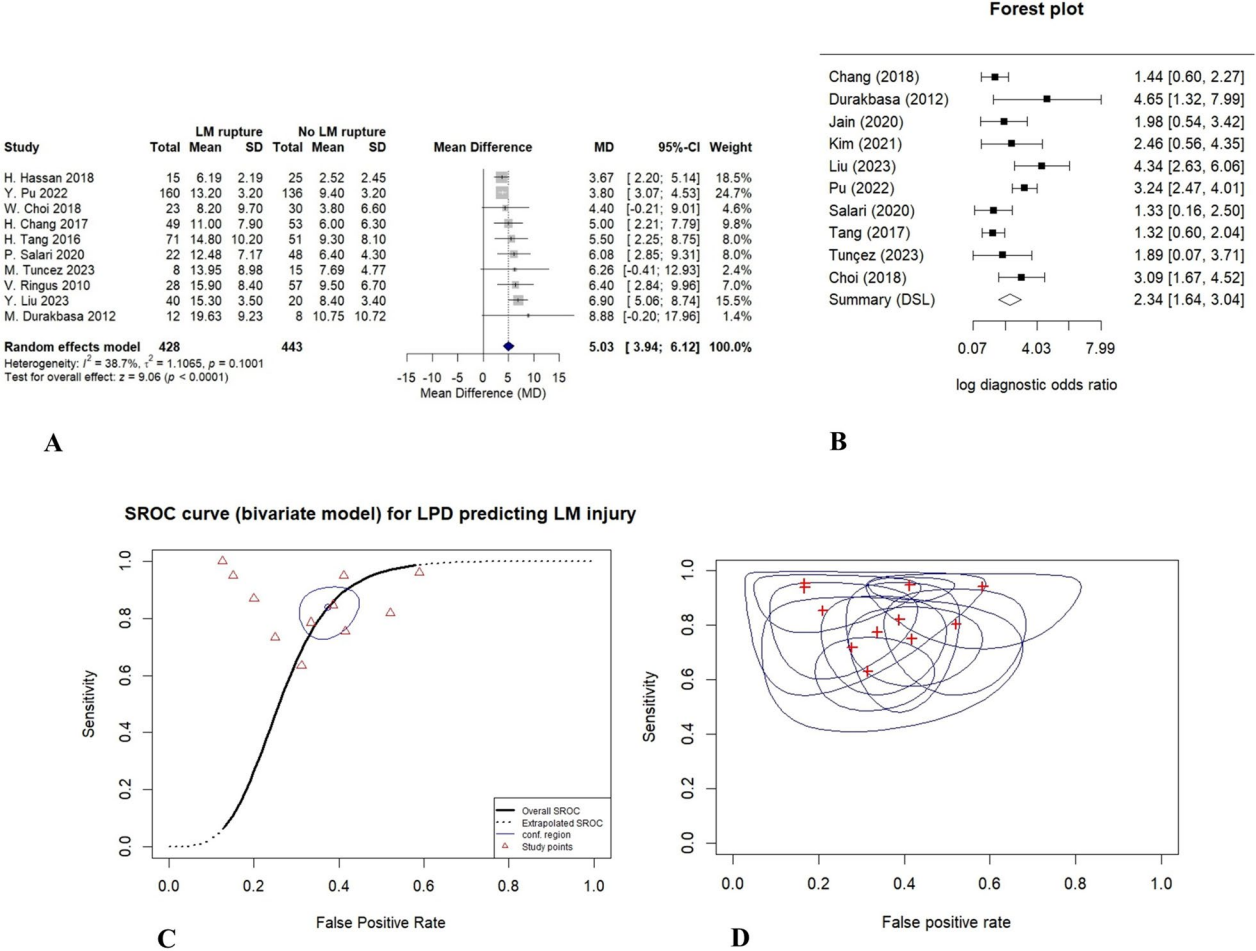
**A** Forest plot illustrating the mean difference (MD) in lateral plateau depression (LPD) between groups with and without lateral meniscus (LM) injury. **B** Univariate forest plot presenting the diagnostic odds ratio (DOR) of LPD for detecting LM injury. **C** Summary receiver operating characteristic (SROC) curve and **D** corresponding ellipse plot derived from the bivariate meta-analysis evaluating the ability of LPD to predict LM injury

A total of 14 studies [5, 16–18, 35–44] evaluated the LPD as a predictive factor for LM injuries. Ten studies [16, 18, 35–40, 42, 43] were eligible for meta-analysis, which demonstrated significantly greater LPD in patients with an LM injury compared with those without an LM injury (MD = 5.03 mm, 95% CI 3.94–6.12), with moderate heterogeneity (*I*^2^ = 38.7%) (Fig. 3A). Subgroup analyses on the basis of reference standard and measurement modality revealed no significant subgroup differences (*P* = 0.107 and *P* = 0.395, respectively) (Supplementary Fig. S1). Sensitivity analysis confirmed the robustness of the findings (Supplementary Fig. S2). Egger’s test revealed evidence of a small-study effect (*P* = 0.033), suggesting potential publication bias (Supplementary Fig. S3). Nevertheless, the trim-and-fill analysis confirmed that the adjusted effect estimate remained significant (MD = 4.08 mm, 95% CI 2.90–5.26) (Supplementary Fig. S4).

Four additional studies [5, 17, 41, 44] were excluded from pooling, because they did not report group-wise means and SDs for LPD, although they provided complementary evidence regarding LM injury. Kim et al. [17] reported that an 11-mm threshold increased the risk of LM tears nine-fold (OR = 9.0, *P* = 0.024), while Spiro et al. [44] observed that each millimeter of depression increased the odds of LM tears by 15% (OR = 1.15, *P* = 0.025). In contrast, Gardner et al. [5] reported no difference in the prevalence of LM tears at an LPD threshold of 6 mm. Wang et al. [41] also found no significant association between LPD and meniscal injury when assessed using CT and X-ray modalities.

For the diagnostic accuracy of LPD in predicting LM injury, the univariate analysis yielded a pooled DOR of 9.80 (95% CI 5.27–18.24; *I*^2^ = 0%) (Fig. 3B). The bivariate meta-analysis indicated that the overall sensitivity was 84% (95% CI 75–90) and the overall specificity was 63% (95% CI 57–68). The total AUC was calculated as 0.724. Heterogeneity was low, with Holling sample-size-adjusted *I*^2^ estimates ranging from 4.0 to 5.6%. The log-likelihood ratio for the goodness-of-fit of the model was 17.79. The SROC and ellipse plot for the bivariate model are presented in Fig. 3C and D, respectively. Deek’s funnel plot analysis revealed no evidence of publication bias (*P* = 0.170) (Supplementary Fig. S5).

#### Medial meniscus

Five studies [5, 17, 35, 36, 41] evaluated the relation between LPD and MM injury. In two studies [5, 35], the LPD was significant in predicting MM injuries with different thresholds: Gardner et al. [5] found a minimal LPD threshold of 8 mm for MM injuries, and Tunçez et al. [35] reported an AUC of 0.882 for predicting MM injuries with LPD < 5.9. Three studies [17, 36, 41] reported that LPD was not significantly associated with MM injuries.

#### Anterior cruciate ligament

**Fig. 4:**
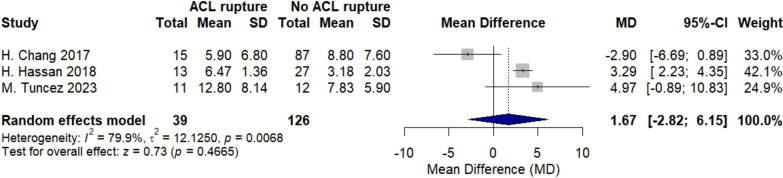
Forest plot illustrating the mean difference (MD) in lateral plateau depression (LPD) between groups with and without anterior cruciate ligament (ACL) injury

The association between ACL injury and LPD was assessed by five papers [5, 35, 36, 38, 44], of which three were suitable for quantitative synthesis [35, 36, 38]. The meta-analysis revealed a nonsignificant difference in the LPD between patients with and without ACL injury (MD = 1.67 mm, 95% CI −2.82 to 6.15), with substantial heterogeneity (*I*^2^ = 79.9%) (Fig. 4). Subgroup analysis based on the reference standard showed a significant subgroup effect (*P* = 0.002), although this was based on a subgroup containing only one arthrotomy/arthroscopy study (Supplementary Fig. S6). Subgroup analysis by measurement modality was not feasible, as all three studies used CT. Sensitivity analysis indicated that the study by Chang et al. [36] was the primary source of heterogeneity, while Egger’s test found no publication bias (*P* = 0.678) (Supplementary Figs. S7-8).

Among excluded reports in quantitative synthesis due to unavailable group‑wise means and SDs, Spiro et al. [44] found the odds of ACL lesions increased by 18% per millimeter of depression (OR = 1.18, *P* = 0.018), whereas Gardner et al. [5] noted no difference in ACL tear prevalence at a depression threshold of 6 mm.

#### Posterior cruciate ligament

Five studies [5, 35, 36, 38, 40] assessed the relationship between LPD and PCL injury. Four studies [5, 35, 36, 40] found no significant association of LPD with PCL injury, and one study [38] reported a 3.11 times higher risk of PCL injury for each millimeter rise in the LPD (OR = 3.11, *P* = 0.030).

#### Collateral ligaments

Four studies [5, 35, 38, 41] reported on the association between LPD and collateral ligament injuries. All except one [41] separated the LCL and MCL. In all of those three [5, 35, 38], LPD had a significant association with LCL injury. No association between LPD and MCL injuries was identified in two studies [5, 35] while another demonstrated a significant relationship (*P* = 0.003) [38]. One study [41] did not separate LCL and MCL tears and reported that collateral injury is predictable with CT (*P* < 0.05).

### LPW in tibial plateau fractures

A total of 13 studies [5, 9, 14, 16, 18, 35–41, 45] measured the LPW as a radiological parameter for predicting soft-tissue injuries, including LM, MM, ACL, PCL, MCL, and LCL.

#### Lateral meniscus

**Fig. 5:**
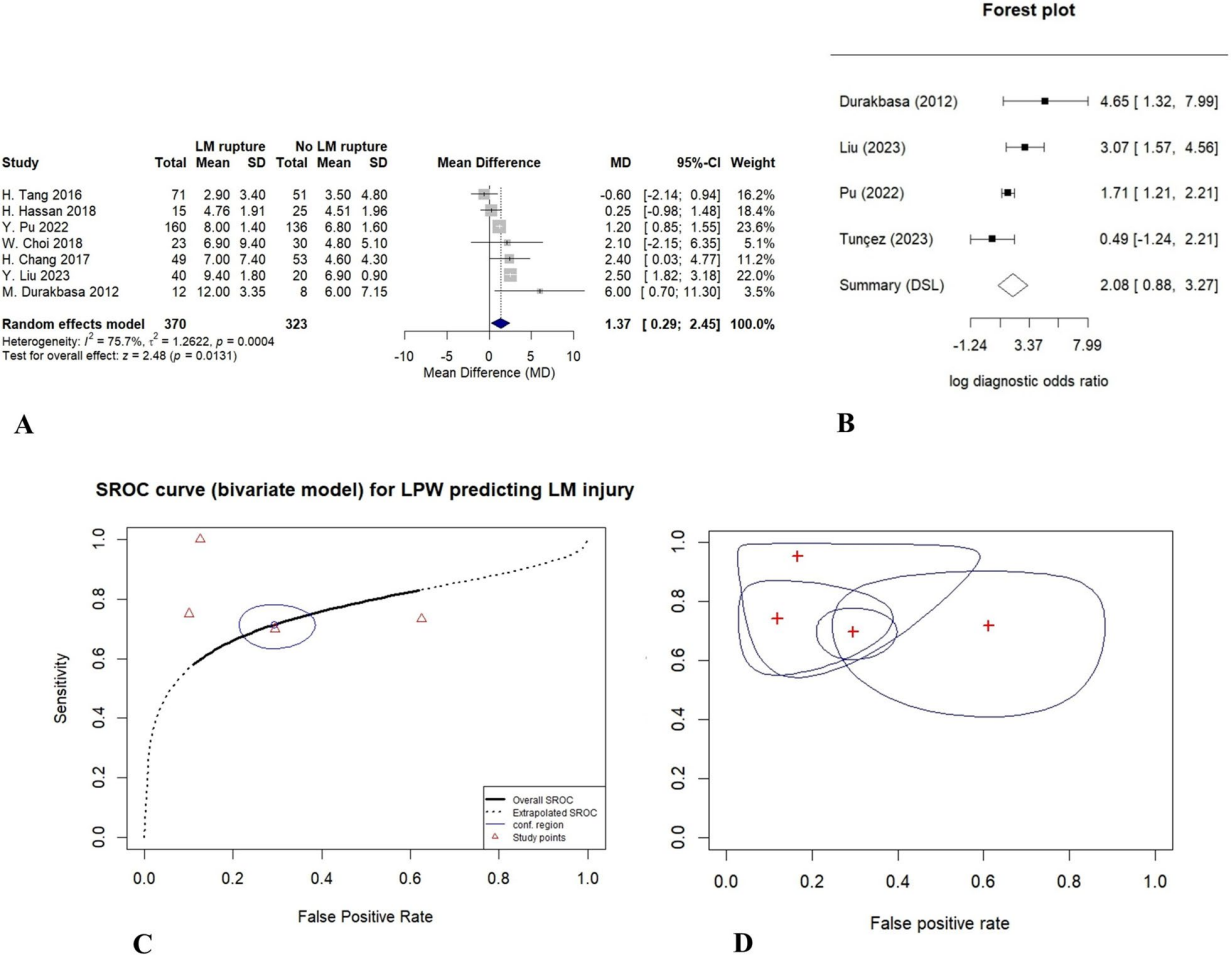
**A** Forest plot illustrating the mean difference (MD) in lateral plateau widening (LPW) between groups with and without lateral meniscus (LM) injury. **B** Univariate forest plot presenting the diagnostic odds ratio (DOR) of LPW for detecting LM injury. **C** Summary receiver operating characteristic (SROC) curve and **D** corresponding ellipse plot derived from the bivariate meta-analysis evaluating the ability of LPW to predict LM injury

A total of 11 studies [5, 14, 16, 18, 35–40] investigated the LPW as a predictor of LM injuries, of which 7 [16, 18, 36–40] were eligible for meta-analysis. The pooled analysis demonstrated a significantly greater LPW in patients with LM injuries compared with those without LM injuries (MD = 1.37 mm, 95% CI 0.29–2.45), although heterogeneity was substantial (*I*^2^ = 75.7%) (Fig. 5A). Subgroup analyses based on reference standard and measurement modality revealed no significant subgroup differences (*P* = 0.186 and *P* = 0.082, respectively) (Supplementary Fig. S9). Leave-one-out sensitivity testing confirmed the stability of results, and Egger’s test indicated no evidence of publication bias (*P* = 0.800) (Supplementary Figs. S10-11).

Four studies [5, 14, 35, 40] excluded from the meta-analysis did not report group-wise means and SDs for LPW, although they provided complementary evidence regarding LM injury. Tang et al. [40] found no significant cutoff for the LPW in predicting LM tears. Kolb et al. [14] reported that each millimeter of plateau widening increased the odds of LM lesions by 40% (OR = 1.40, *P* = 0.021). Tunçez et al. [35] observed that plateau widening greater than 4.2 mm was more predictive of LCL tears than LM tears. Gardner et al. [5] further noted that a combined plateau depression > 6 mm and widening > 5 mm markedly elevated the LM tear risk.

For the diagnostic accuracy of LPW in predicting LM injury, the univariate analysis showed a pooled DOR of 7.971 (95% CI 2.42–26.32; *I*^2^ = 28.2%) (Fig. 5B). The bivariate meta-analysis indicated that the overall sensitivity was 71% (95% CI 65–77) and the overall specificity was 71% (95% CI 63–77). The total AUC was 0.758. Heterogeneity was low, with Holling sample-size-adjusted *I*^2^ estimates ranging from 2.7 to 4%. The log-likelihood ratio for the goodness-of-fit of the model was 6.69. The SROC and ellipse plot for the bivariate model are presented in Fig. 5C and D, respectively. Deek’s funnel plot analysis revealed no evidence of publication bias (*P* = 0.536) (Supplementary Fig. S12).

#### Medial meniscus

Three studies [5, 17, 36] investigated the association between MM injuries and the LPW. Of these, two [17, 36] reported no diagnostic value of LPW for MM injuries, while one study [5] found a significant association between LPW and MM tears (*P* = 0.006).

#### Anterior cruciate ligament

**Fig. 6:**
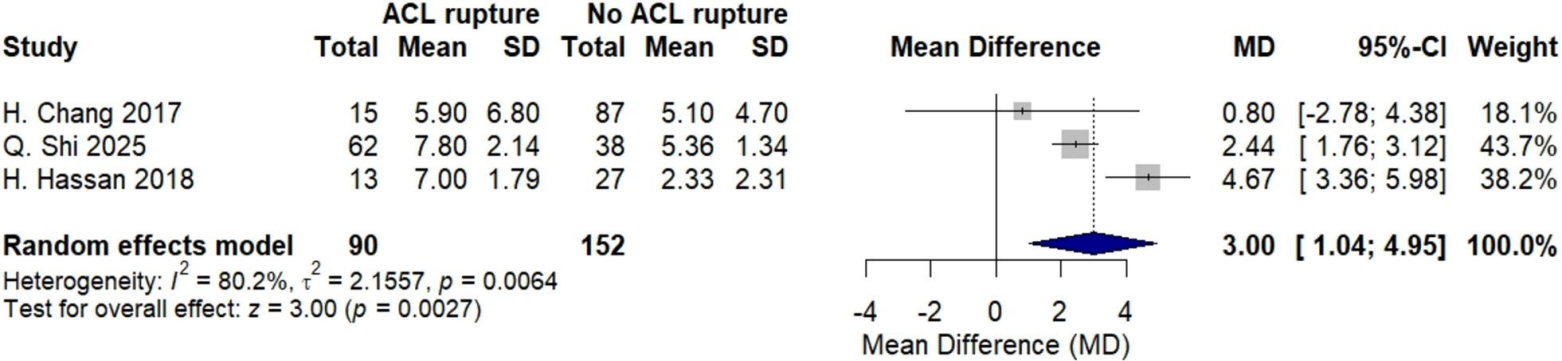
Forest plot illustrating the mean difference (MD) in lateral plateau widening (LPW) between groups with and without anterior cruciate ligament (ACL) injury

Five studies [5, 35, 36, 38, 45] evaluated LPW as a predictor of ACL injuries, of which three studies [36, 38, 45] were included in the meta-analysis. The pooled analysis demonstrated that patients with ACL injuries had significantly greater LPW than those without ACL injuries (MD = 3.00 mm, 95% CI 1.04–4.95), although substantial heterogeneity was observed (*I*^2^ = 80.2%) (Fig. 6). Subgroup analysis based on the reference standard revealed a significant subgroup effect (*P* = 0.002); however, this finding was derived from a subgroup containing only one MRI-based study. In contrast, subgroup analysis according to measurement modality showed no significant difference (*P* = 0.729) (Supplementary Fig. S13). Leave‑one‑out sensitivity analysis indicated that the study by Hassan et al. [38] was the primary source of heterogeneity, and Egger’s test demonstrated no evidence of publication bias (*P* = 0.911) (Supplementary Figs. S14–15).

Two studies [5, 35] were not included in the quantitative synthesis due to the absence of group‑wise means and SDs for LPW, though both contributed relevant data on ACL injury. Gardner et al. [5] found no significant difference in ACL tear prevalence at an LPW threshold of 9 mm. At a threshold of 8 mm, Tunçez et al. [35] reported a poor sensitivity of 36% and specificity of 83%.

#### Posterior cruciate ligament and collateral ligaments

PCL and collateral ligament injuries were evaluated in six papers [5, 14, 35, 36, 38, 41]. The LPW was significantly elevated in the LCL injury group [5, 14, 38], with one study reporting a sensitivity of 100% and specificity of 81% for this parameter [35]. A meaningful association with PCL tears was reported in two studies [5, 38].

### Other predictors for soft-tissue injury

Additional radiological parameters were assessed for predicting soft-tissue injuries [36, 43, 45]. Joint fluid accumulation was significantly associated with ACL injury (*P* = 0.005) [45]. Medial plateau widening (MPW) and medial plateau depression (MPD) were significantly associated with PCL and MM lesions [36]. Salari et al. [43] reported that articular depression in different columns of the tibial plateau was significantly associated with meniscal injury.

## Discussion

Our systematic review and meta-analysis indicate that radiographic parameters, particularly LPD and LPW, may serve as useful predictors of ligament and meniscal injuries in lateral tibial plateau fractures. Across 17 studies encompassing 1348 patients, both the LPD and LPW demonstrated significant associations with LM injuries, with pooled analyses confirming moderate diagnostic accuracy (AUC 0.724 for LPD and 0.758 for LPW). While a pooled MD of 5 mm in depression provided a notable difference between patients with and without LM injuries, the 1.37 mm widening difference was relatively small and may represent a statistically detectable but clinically modest effect, consistent with the substantial heterogeneity observed in LPW analyses. Beyond meniscal injury, LPW was also significantly associated with ACL injuries (MD = 3.00 mm), and LPD showed more consistent associations with LCL injury. However, the findings for ligamentous injuries were generally more heterogeneous than those for LM injuries.

The LM demonstrated the most reproducible association with radiographic displacement parameters in tibial plateau fractures, particularly with LPD. Patients with LM injuries consistently exhibited approximately 5 mm greater depression than those without injuries, with pooled sensitivity and specificity values of 84% and 63%, respectively. These findings support the concept that axial loading and subchondral articular collapse directly transmit compressive forces to the LM and its capsular attachments, reinforcing the role of LPD as a marker of LM injury [46]. Threshold-dependent relationships further reinforce this association: Kim et al. [17] reported that depression greater than 11 mm increased the odds of LM tears nine-fold, whereas Spiro et al. [44] demonstrated a 15% increase in tear risk for every additional millimeter of depression. LPW was also associated with LM injuries, although the pooled widening difference was smaller than that observed for depression (1.37 versus 5 mm). Kolb et al. [14] found that each additional millimeter of widening increased the odds of LM lesions by 40%. Notably, Gardner et al. [5] reported significant increased tear risk when widening and depression were combined. It was also reported that peripheral meniscal injuries may occur even in minimally depressed fractures, suggesting that shear and varus/valgus stresses may also contribute to LM pathology [41].

LPD and LPW may represent distinct biomechanical mechanisms rather than interchangeable measures of fracture severity. LPD appears to primarily reflect axial compressive loading transmitted through the femoral condyles into the subchondral bone and meniscal attachments, which likely contributes to its more consistent association with LM injury [47]. In contrast, LPW may better represent shear, translational, and valgus-directed instability across the knee joint. Tang et al. [40] proposed that plateau widening may incompletely reflect the true injury energy in complex fracture patterns because multidirectional displacement, including anterior or posterior translation of the lateral plateau, may occur even in the presence of minimal widening. In contrast, articular depression may more directly represent compressive loading transmitted to the lateral meniscus. Chang et al. [36] likewise hypothesized that LPW reflects the magnitude of valgus stress applied across the knee, whereas LPD more directly represents compressive articular collapse. This biomechanical distinction may explain why LPD demonstrated a more reproducible association with LM injury in comparison with LPW in our pooled analyses, despite both parameters being significantly associated with LM injuries.

In contrast to the consistent findings for LM injury, evidence regarding other meniscal and ligamentous injuries was substantially more heterogeneous. Cruciate and collateral ligament injuries likely result from more complex multidirectional loading mechanisms involving rotational, shear, hyperextension, and valgus/varus forces [48, 49]. Consistent with this, our pooled analysis demonstrated no significant association between LPD and ACL injuries despite isolated positive findings from Hassan et al. [38] and Spiro et al. [44]. In contrast, LPW demonstrated a significant pooled association with ACL injury, supporting the hypothesis that widening may better reflect valgus and translational instability patterns involved in ACL trauma [50, 51].

Furthermore, associations between LPD or LPW and medial meniscal injury varied considerably across studies, with some reports identifying predictive thresholds [5, 35] and others finding no meaningful relationship [17, 36, 41]. Although several studies linked LPD and LPW to LCL or PCL injuries [5, 14, 35, 38], these associations remained less consistent than those observed for LM pathology. An MRI-based study by Kode et al. [52] also demonstrated correlations between increasing depression depth and both meniscal and cruciate injuries, although the relationships were weaker and less reproducible than those involving the lateral meniscus.

Several additional radiographic parameters have also been investigated as markers of soft-tissue injury. The MPD and MPW for medial tibial plateau fractures were reported by Chang et al. [36] as predictors of MM lesions, highlighting that displacement in the medial compartment carries its own risk. Synovial fluid volume has also been linked to cruciate injuries, with Hassan et al. [38] demonstrating a significant correlation between joint effusion and ACL lesions. Salari et al. [43] further emphasized the importance of fracture morphology, showing that anterior column fractures were associated with up to seven-fold greater odds of meniscal tears compared with posterior column fractures. In addition, Tunçez et al. [35] reported that mild LPD (< 5.9 mm) was associated with MM tears, whereas more severe depression (> 12 mm) was linked specifically to bucket-handle tears of the lateral meniscus. Collectively, these findings suggest that soft-tissue injury patterns in tibial plateau fractures are multifactorial and that integrating additional radiographic features with LPD and LPW may further improve risk stratification and surgical planning.

Clinically, LPD and LPW should be viewed as adjunctive risk markers rather than standalone diagnostic tools. Increased values should heighten preoperative suspicion for associated soft-tissue pathology, support selective MRI use in patients with substantial displacement or equivocal clinical findings, and prompt more careful intraoperative assessment of the menisci and ligaments. Given the moderate diagnostic accuracy observed in our analyses, these radiographic parameters are best interpreted as tools for risk stratification rather than substitutes for direct diagnostic evaluation.

A key consideration in interpreting our findings is the variability in imaging modalities and in the reference standards used for soft-tissue injury confirmation. It has been hypothesized that CT may improve agreement by providing more accurate quantification of articular displacement [53]. Johannsen et al. [54] showed that radiographic and CT-based measurements of tibial plateau depression are not directly interchangeable; however, they found that AP radiographs and coronal CT may yield broadly comparable estimates of fracture widening. Similarly, Wang et al. [41] reported that while both radiography and CT can predict soft-tissue injury, their threshold values differ. In addition, reference standards for diagnosing soft-tissue injuries varied across studies, including MRI, arthroscopy, arthrotomy, or their combinations. While arthroscopy is considered the most direct assessment of intra-articular pathology, MRI accuracy may be influenced by factors such as acute edema, hemarthrosis, and imaging timing [13, 55]. Nonetheless, the subgroup analyses in current review did not demonstrate major differences across modalities or reference standards; however, these methodological variations likely contributed to the observed heterogeneity.

This study has several limitations that should be acknowledged. Many included studies did not provide complete descriptive statistics for injured and noninjured groups, often reporting only threshold values or cutoff points. In some analyses, means and SDs had to be estimated from medians, ranges, or IQRs, which may have reduced precision. In addition, variability in imaging modalities, measurement techniques, and reference standards for soft-tissue injury confirmation likely contributed to heterogeneity across pooled estimates. Substantial heterogeneity was observed in several analyses, particularly for LPW–LM and LPD–ACL associations. Although trim-and-fill analysis did not materially alter the conclusions, evidence of a small-study effect was identified in the LPD–LM continuous meta-analysis. Furthermore, the evidence base for non-LM outcomes was comparatively limited and less consistent than that for LM injuries. Finally, most included studies were retrospective cohorts or case series, making them susceptible to selection bias and limiting causal inference.

In conclusion, LPD and LPW appear to be useful adjunctive radiographic markers for predicting soft-tissue injury in tibial plateau fractures. LPD showed the most consistent association with LM injuries, with approximately 5 mm greater depression in injured patients. LPW also demonstrated a smaller and more variable association with LM injury, with a pooled mean difference of 1.37 mm. While LPW was significantly associated with ACL injury, the evidence for ACL and other ligamentous injuries was more limited and heterogeneous than that for LM injury. When interpreted alongside fracture characteristics, these routinely available imaging measures can aid in risk stratification and support targeted use of MRI or careful intraoperative assessment to optimize meniscal and ligament preservation.

## Supplementary Information

Supplementary material 1.

## Data Availability

The data used and/or analyzed during the current study are available from the corresponding author on reasonable request.

## Abbreviations

MRI: Magnetic resonance imaging
LPD: Lateral plateau depression
LPW: Lateral plateau widening
LM: Lateral meniscus
MM: Medial meniscus
DTA: Diagnostic test accuracy
DOR: Diagnostic odds ratio
ROC: Receiver operating characteristic
