# Supplementary material for: Tibial plateau depression and widening as predictors of meniscal and ligamentous injuries: a systematic review and meta-analysis
Source: Knee Surg Relat Res. 2026 Jul 6;38:25. doi: 10.1186/s43019-026-00332-6 (PMC13339280; doi:10.1186/s43019-026-00332-6)
Supplement: Supplementary file 1 — Supplementary material 1. [file 43019_2026_332_MOESM1_ESM.docx]

**Table S1.** Complete search query in databases

| Database | Search query | Number of results |
| --- | --- | --- |
| PubMed | (Menisc* injur*[Title/Abstract] OR Menisc* tear[Title/Abstract] OR Menisc* contusion[Title/Abstract] OR Cruciate ligament injur*[Title/Abstract] OR ACL injur*[Title/Abstract] OR PCL injur*[Title/Abstract] OR ACL avulsion fracture[Title/Abstract] OR ACL partial tear[Title/Abstract] OR ACL complete tear[Title/Abstract] OR PCL avulsion fracture[Title/Abstract] OR PCL tear[Title/Abstract] OR intra-articular soft tissue injur*[Title/Abstract] OR soft-tissue complication[Title/Abstract] OR Lateral meniscal injur*[Title/Abstract] OR Menisc* tear[Title/Abstract] OR Peripheral menisc* detachment[Title/Abstract] OR Meniscocapsular junction[Title/Abstract] OR Menisc* entrapment[Title/Abstract] OR Lateral menisc* retraction[Title/Abstract] OR Menisc* lesion[Title/Abstract] OR Ligament injur*[Title/Abstract] OR MCL injur*[Title/Abstract] OR LCL injur*[Title/Abstract] OR Lateral collateral ligament injur*[Title/Abstract] OR Medial collateral ligament injur*[Title/Abstract] OR Flap Tears[Title/Abstract] OR Soft Tissue Injuries[MeSH Terms] OR Tibial Meniscus Injuries[MeSH Terms] OR Anterior Cruciate Ligament Injuries[MeSH Terms]) AND (lateral plateau depression[Title/Abstract] OR lateral joint depression[Title/Abstract] OR LPD[Title/Abstract] OR medial plateau depression[Title/Abstract] OR medial joint depression[Title/Abstract] OR MPD[Title/Abstract] OR lateral plateau widening[Title/Abstract] OR lateral joint widening[Title/Abstract] OR LPW[Title/Abstract] OR medial plateau widening[Title/Abstract] OR medial joint widening[Title/Abstract] OR MPW[Title/Abstract] OR intra-articular step-off[Title/Abstract] OR articular depression[Title/Abstract] OR plateau displacement[Title/Abstract] OR articular widening[Title/Abstract] OR joint depression[Title/Abstract] OR plateau collapse[Title/Abstract] OR widening[Title/Abstract] OR mediolateral width[Title/Abstract] OR splaying[Title/Abstract] OR Gap[Title/Abstract]) AND (Tibia* plateau fracture[Title/Abstract] OR Metaphyseal fracture[Title/Abstract] OR Bicondylar Tibial Plateau Fracture[Title/Abstract] OR Tibial Plateau Fractures[MeSH Terms] OR Lateral Tibial Plateau Fracture[Title/Abstract] OR Medial Tibial Plateau Fracture[Title/Abstract]) | 21 |
| Scopus | TITLE-ABS-KEY("Meniscal injury" OR "Meniscus tear" OR "Meniscus contusion" OR "Cruciate ligament injury" OR "ACL injury" OR "PCL injury" OR "ACL avulsion fracture" OR "ACL partial tear" OR "ACL complete tear" OR "PCL avulsion fracture" OR "PCL tear" OR "intra-articular soft tissue injury" OR "soft-tissue complication" OR "Lateral meniscal injury" OR "Peripheral meniscal detachment" OR "Meniscocapsular junction" OR "Meniscal entrapment" OR "Lateral meniscus retraction" OR "Meniscal lesion" OR "Ligament injury" OR "MCL injury" OR "LCL injury" OR "Lateral collateral ligament injury" OR "Medial collateral ligament injury" OR "Flap tear" OR "Soft Tissue Injuries" OR "Tibial Meniscus Injuries" OR "Anterior Cruciate Ligament Injuries") AND TITLE-ABS-KEY("lateral plateau depression" OR "lateral joint depression" OR "LPD" OR "medial plateau depression" OR "medial joint depression" OR "MPD" OR "lateral plateau widening" OR "lateral joint widening" OR "LPW" OR "medial plateau widening" OR "medial joint widening" OR "MPW" OR "intra-articular step-off" OR "articular depression" OR "plateau displacement" OR "widening" OR "mediolateral width" OR "splaying" OR "Gap" OR "articular widening" OR "joint depression" OR "plateau collapse") AND TITLE-ABS-KEY("Tibial plateau fracture" OR "Metaphyseal fracture" OR "Bicondylar Tibial Plateau Fracture" OR "Lateral Tibial Plateau Fracture" OR "Medial Tibial Plateau Fracture") | 40 |
| Embase | ('meniscal injury':ti,ab,kw OR 'meniscus tear':ti,ab,kw OR 'meniscus contusion':ti,ab,kw OR 'cruciate ligament injury':ti,ab,kw OR 'acl injury':ti,ab,kw OR 'pcl injury':ti,ab,kw OR 'acl avulsion fracture':ti,ab,kw OR 'acl partial tear':ti,ab,kw OR 'acl complete tear':ti,ab,kw OR 'pcl avulsion fracture':ti,ab,kw OR 'pcl tear':ti,ab,kw OR 'intra-articular soft tissue injury':ti,ab,kw OR 'soft-tissue complication':ti,ab,kw OR 'lateral meniscal injury':ti,ab,kw OR 'peripheral meniscal detachment':ti,ab,kw OR 'meniscocapsular junction':ti,ab,kw OR 'meniscal entrapment':ti,ab,kw OR 'lateral meniscus retraction':ti,ab,kw OR 'meniscal lesion':ti,ab,kw OR 'ligament injury':ti,ab,kw OR 'mcl injury':ti,ab,kw OR 'Flap tear':ti,ab,kw OR 'lcl injury':ti,ab,kw OR 'lateral collateral ligament injury':ti,ab,kw OR 'medial collateral ligament injury':ti,ab,kw OR 'soft tissue injuries':ti,ab,kw OR 'tibial meniscus injuries':ti,ab,kw OR 'anterior cruciate ligament injuries':ti,ab,kw) AND ('lateral plateau depression':ti,ab,kw OR 'lateral joint depression':ti,ab,kw OR 'lpd':ti,ab,kw OR 'medial plateau depression':ti,ab,kw OR 'medial joint depression':ti,ab,kw OR 'mpd':ti,ab,kw OR 'lateral plateau widening':ti,ab,kw OR 'lateral joint widening':ti,ab,kw OR 'lpw':ti,ab,kw OR 'medial plateau widening':ti,ab,kw OR 'medial joint widening':ti,ab,kw OR 'mpw':ti,ab,kw OR 'intra-articular step-off':ti,ab,kw OR 'articular depression':ti,ab,kw OR 'plateau displacement':ti,ab,kw OR 'articular widening':ti,ab,kw OR 'joint depression':ti,ab,kw OR 'plateau collapse':ti,ab,kw OR 'Gap':ti,ab,kw OR 'splaying':ti,ab,kw OR 'mediolateral width ':ti,ab,kw OR 'widening':ti,ab,kw OR 'tibial plateau fractures':ti,ab,kw) AND ('tibial plateau fracture':ti,ab,kw OR 'metaphyseal fracture':ti,ab,kw OR 'Bicondylar Tibial Plateau Fracture':ti,ab,kw OR 'Lateral Tibial Plateau Fracture':ti,ab,kw OR 'Medial Tibial Plateau Fracture':ti,ab,kw) | 83 |
| Web of Science | (TS=("Meniscal injury") OR TS=("Meniscus tear") OR TS=("Meniscus contusion") OR TS=("Cruciate ligament injury") OR TS=("ACL injury") OR TS=("PCL injury") OR TS=("ACL avulsion fracture") OR TS=("ACL partial tear") OR TS=("Flap tear") OR TS=("ACL complete tear") OR TS=("PCL avulsion fracture") OR TS=("PCL tear") OR TS=("intra-articular soft tissue injury") OR TS=("soft-tissue complication") OR TS=("Lateral meniscal injury") OR TS=("Peripheral meniscal detachment") OR TS=("Meniscocapsular junction") OR TS=("Meniscal entrapment") OR TS=("Lateral meniscus retraction") OR TS=("Meniscal lesion") OR TS=("Ligament injury") OR TS=("MCL injury") OR TS=("LCL injury") OR TS=("Lateral collateral ligament injury") OR TS=("Medial collateral ligament injury") OR TS=("Soft Tissue Injuries") OR TS=("Tibial Meniscus Injuries") OR TS=("Anterior Cruciate Ligament Injuries"))AND(TS=("lateral plateau depression") OR TS=("lateral joint depression") OR TS=("LPD") OR TS=("medial plateau depression") OR TS=("medial joint depression") OR TS=("MPD") OR TS=("lateral plateau widening") OR TS=("lateral joint widening") OR TS=("LPW") OR TS=("medial plateau widening") OR TS=("medial joint widening") OR TS=("MPW") OR TS=("widening") OR TS=("mediolateral width") OR TS=("splaying") OR TS=("Gap") OR TS=("intra-articular step-off") OR TS=("articular depression") OR TS=("plateau displacement") OR TS=("articular widening") OR TS=("joint depression") OR TS=("plateau collapse") OR) AND (TS=("Tibial plateau fracture") OR TS=("Metaphyseal fracture") OR TS=("Bicondylar Tibial Plateau Fracture") OR TS=("Medial Tibial Plateau Fracture") OR TS=("Letral Tibial Plateau Fracture")) | 18 |

**
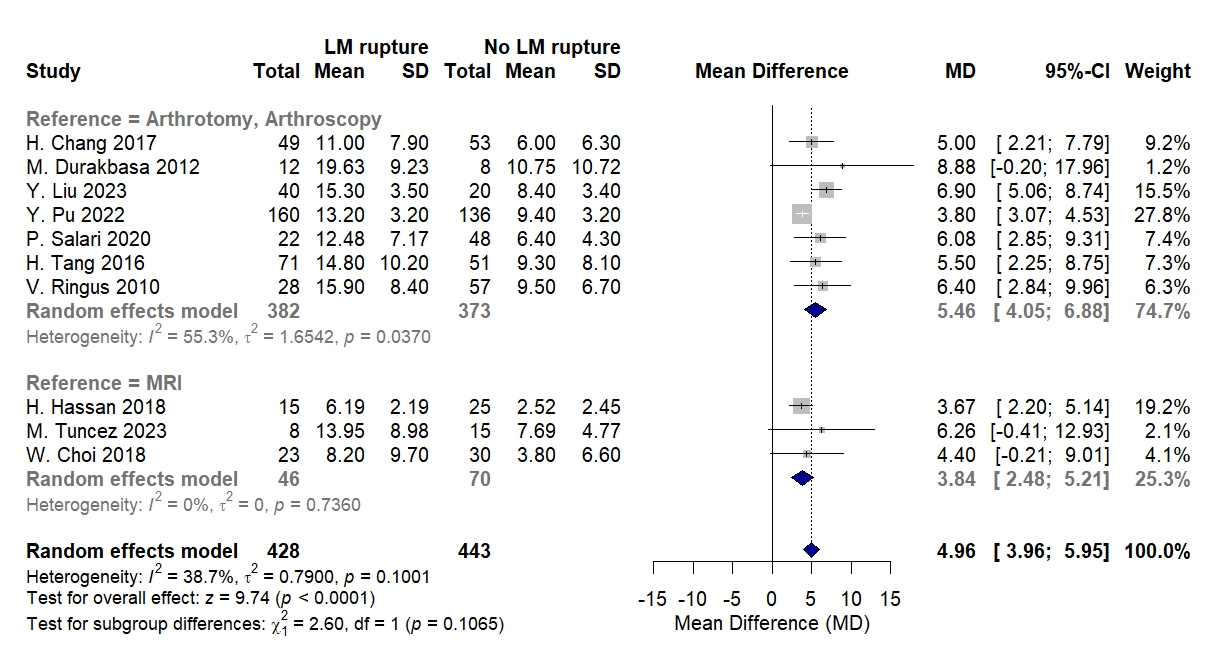
**

**A**

**
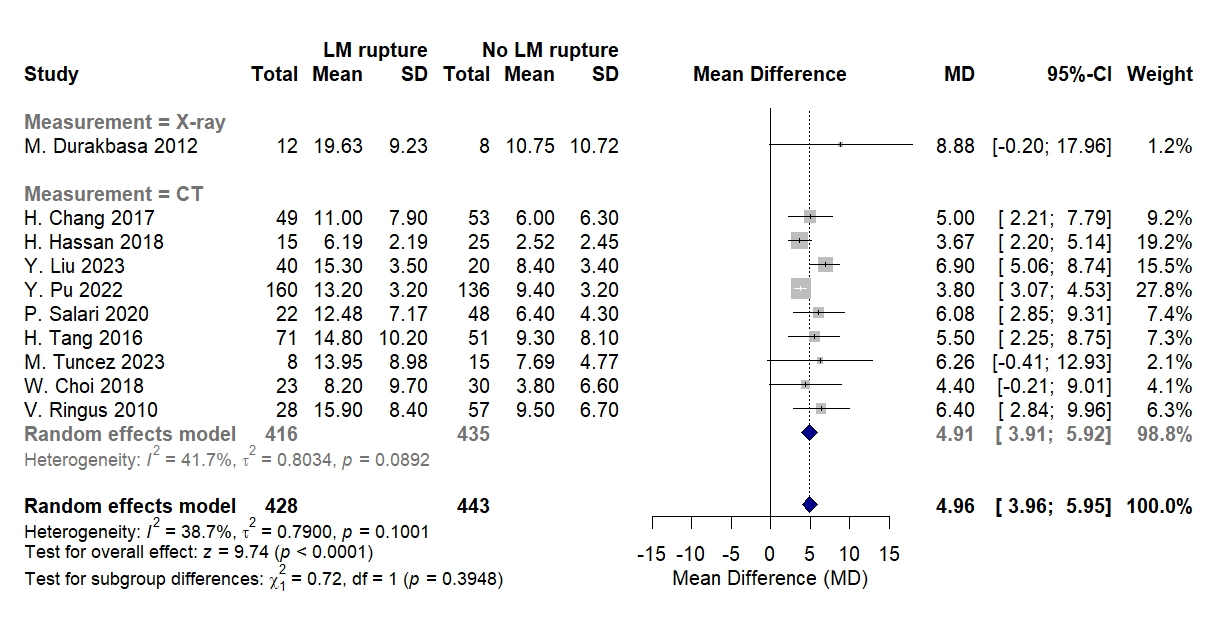
**

**B**

**Figure S1.** Subgroup analysis of the meta-analysis assessing mean difference (MD) in lateral plateau depression (LPD) between groups with and without lateral meniscus (LM) injury based on (A) reference standard and (B) measurement modality

**
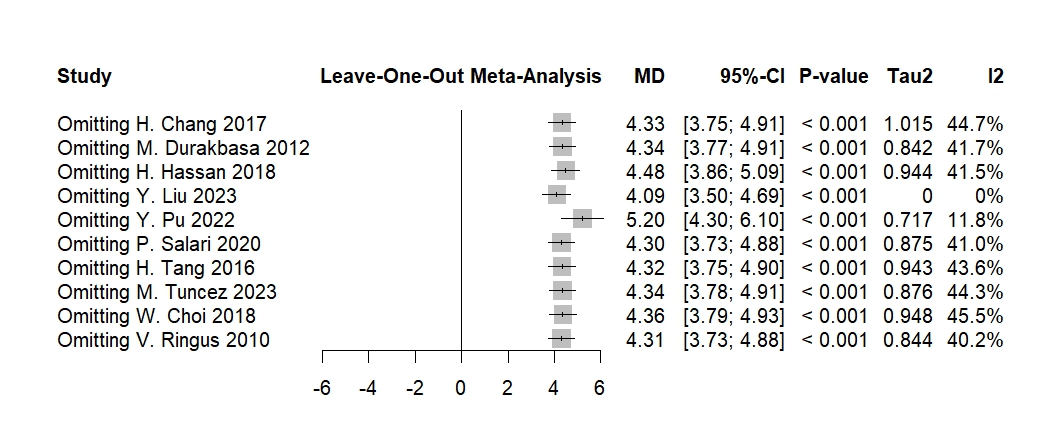
**

**Figure S2.** Leave-one-out sensitivity analysis of the meta-analysis assessing mean difference (MD) in lateral plateau depression (LPD) between groups with and without lateral meniscus (LM) injury


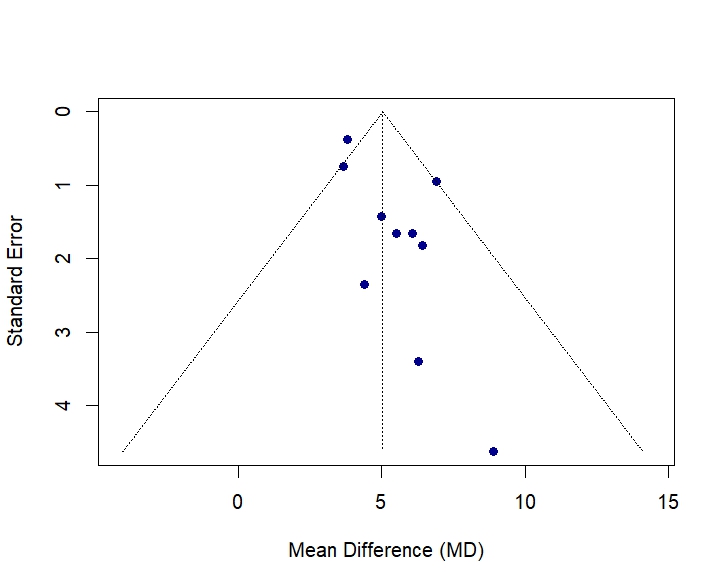


**Figure S3.** Funnel plot of mean difference (MD) in lateral plateau depression (LPD) between groups with and without lateral meniscus (LM) injury

**
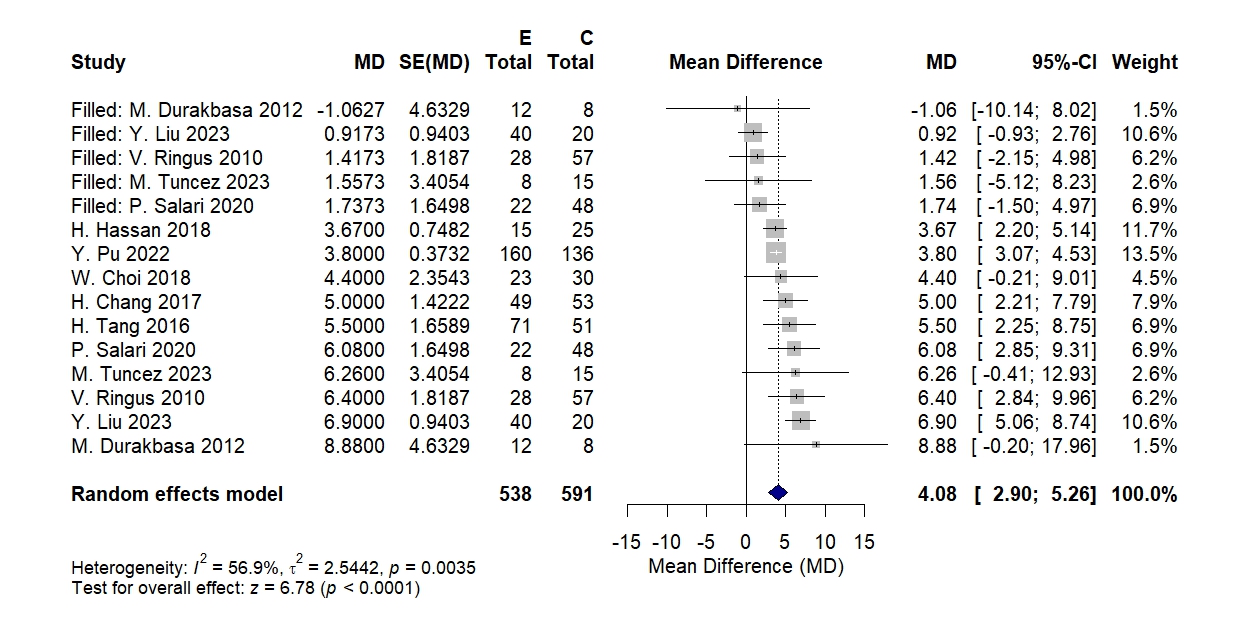
**

**Figure S4.** Trim-and-fill plot of mean difference (MD) in lateral plateau depression (LPD) between groups with and without lateral meniscus (LM) injury

**
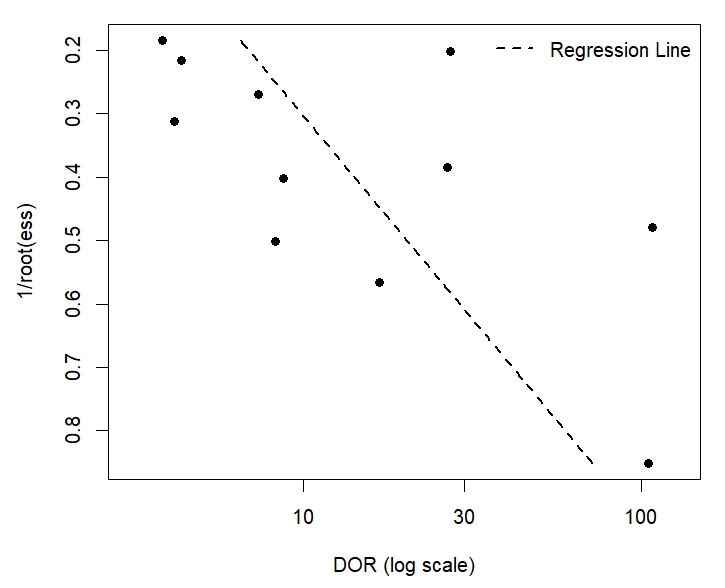
**

**Figure S5.** Deek’s funnel plot of bivariate model of lateral plateau depression (LPD) predicting lateral meniscus (LM) injury


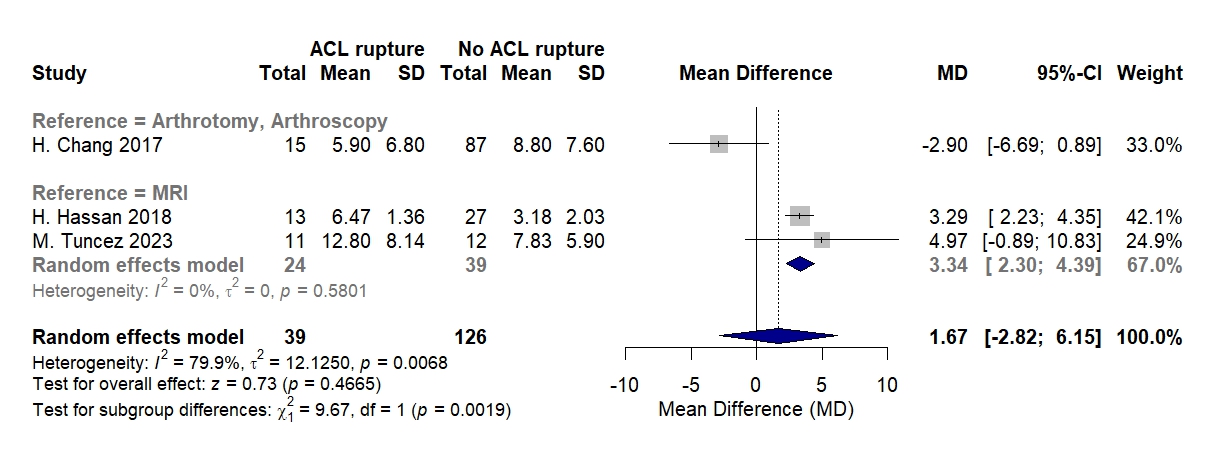


**Figure S6.** Subgroup analysis of the meta-analysis assessing mean difference (MD) in lateral plateau depression (LPD) between groups with and without anterior cruciate ligament (ACL) injury based on reference standard

**
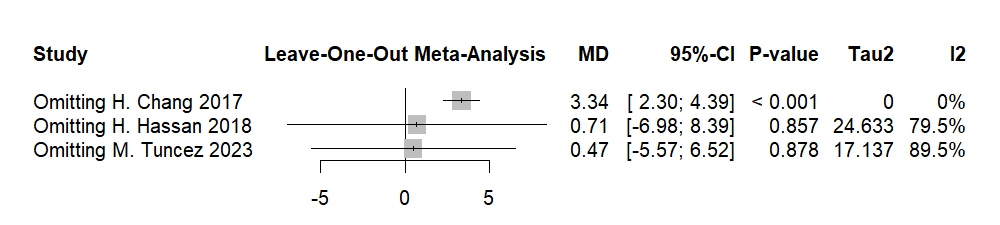
Figure S7.** Leave-one-out sensitivity analysis of the meta-analysis assessing mean difference (MD) in lateral plateau depression (LPD) between groups with and without anterior cruciate ligament (ACL) injury


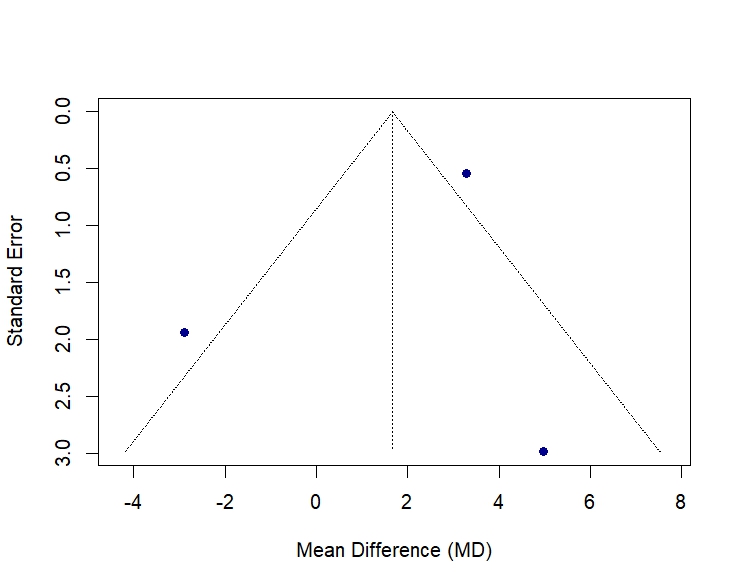


**Figure S8.** Funnel plot of mean difference (MD) in lateral plateau depression (LPD) between groups with and without anterior cruciate ligament (ACL) injury


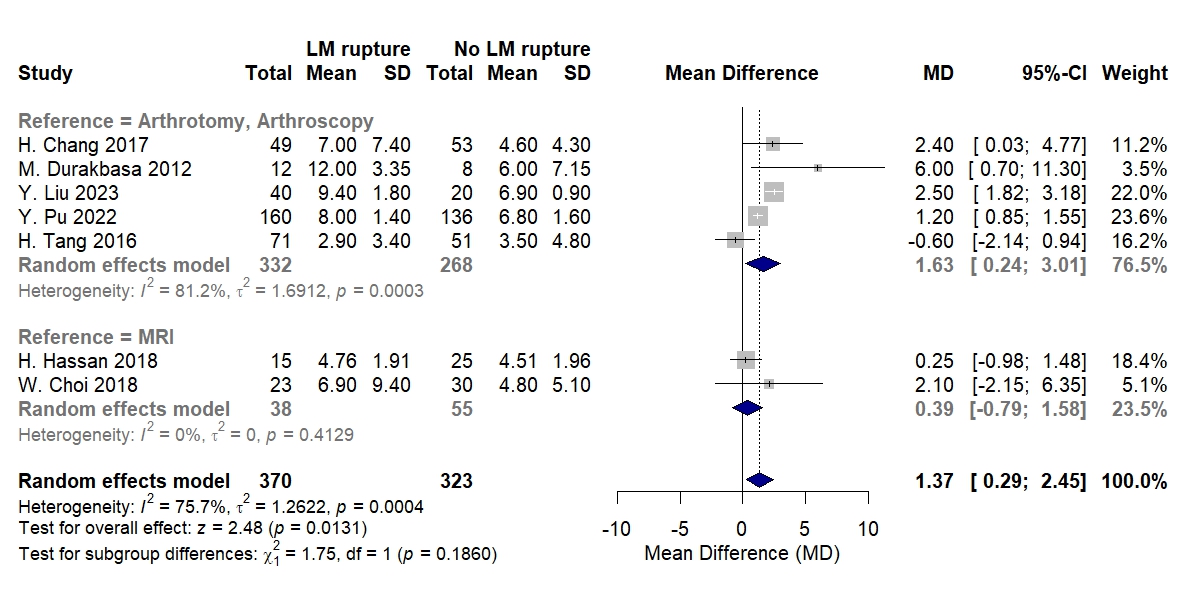


**A**


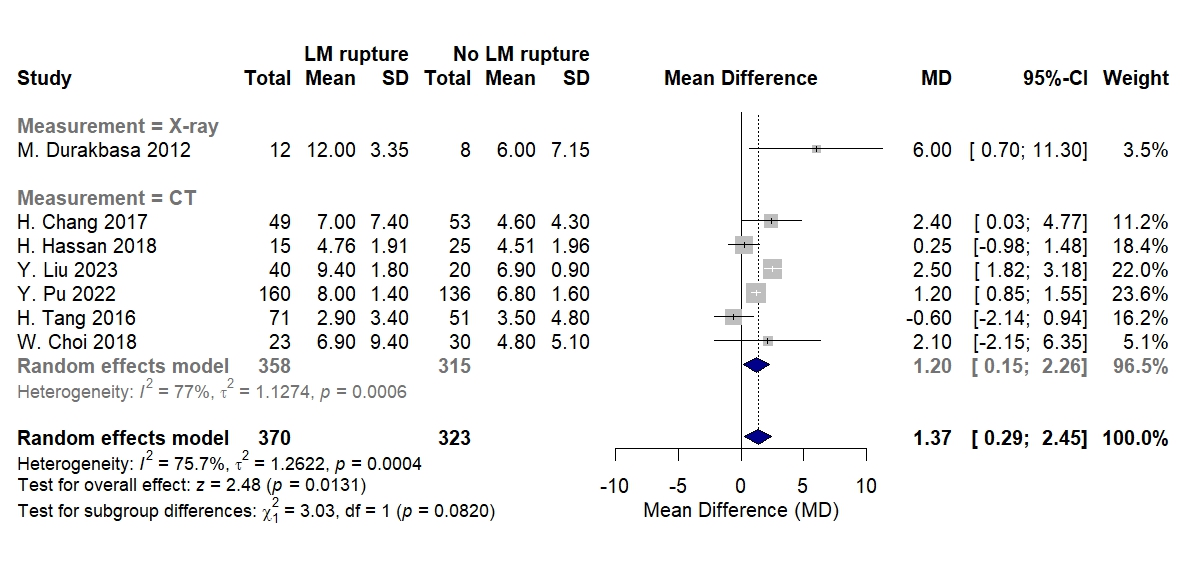


**B**

**Figure S9.** Subgroup analysis of the meta-analysis assessing mean difference (MD) in lateral plateau widening (LPW) between groups with and without lateral meniscus (LM) injury based on (A) reference standard and (B) measurement modality


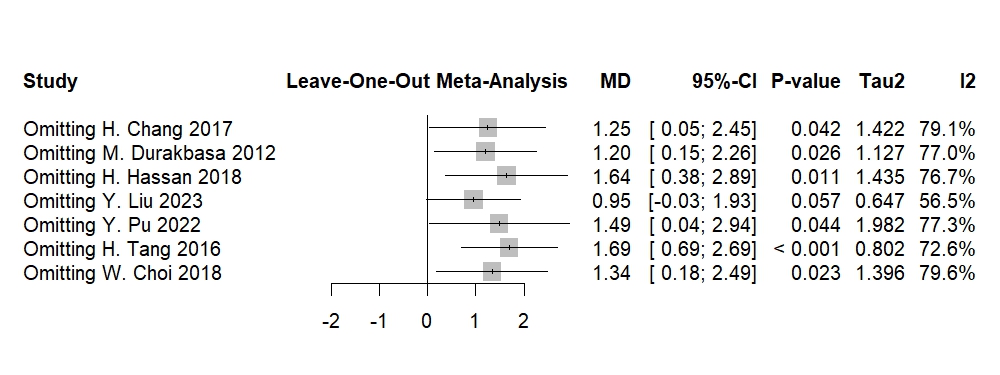
**Figure S10.** Leave-one-out sensitivity analyses of the meta-analysis assessing mean difference (MD) in lateral plateau widening (LPW) between groups with and without lateral meniscus (LM) injury


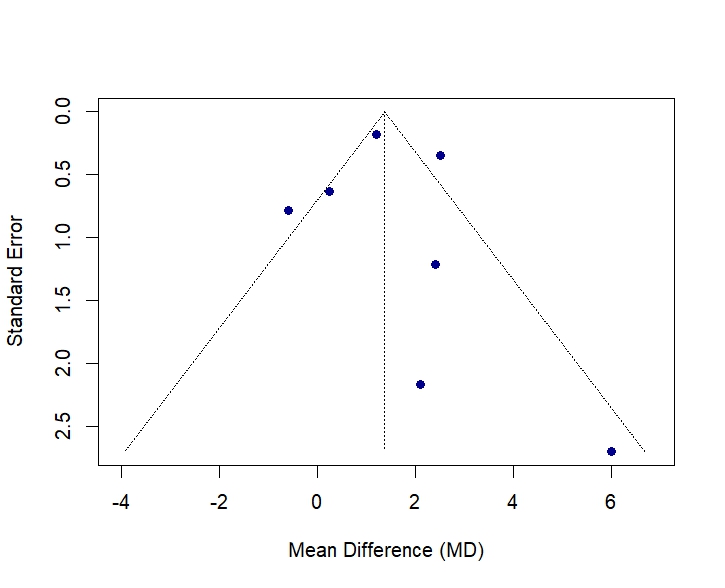


**Figure S11.** Funnel plot of mean difference (MD) in lateral plateau widening (LPW) between groups with and without lateral meniscus (LM) injury


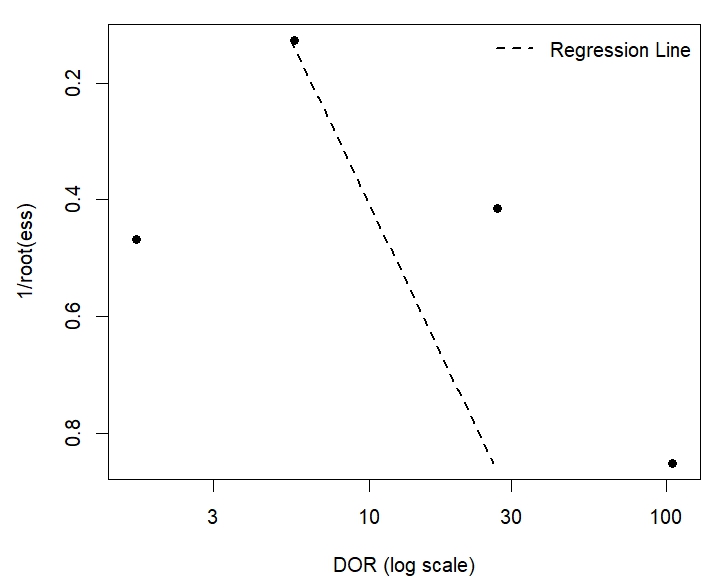


**Figure S12.** Deek’s funnel plot of bivariate model of lateral plateau widening (LPW) predicting lateral meniscus (LM) injury


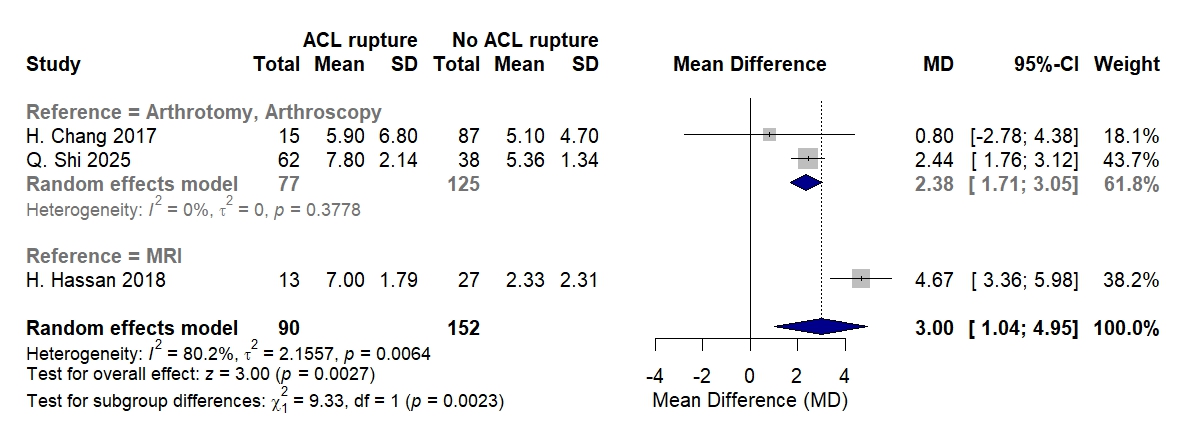


**A**


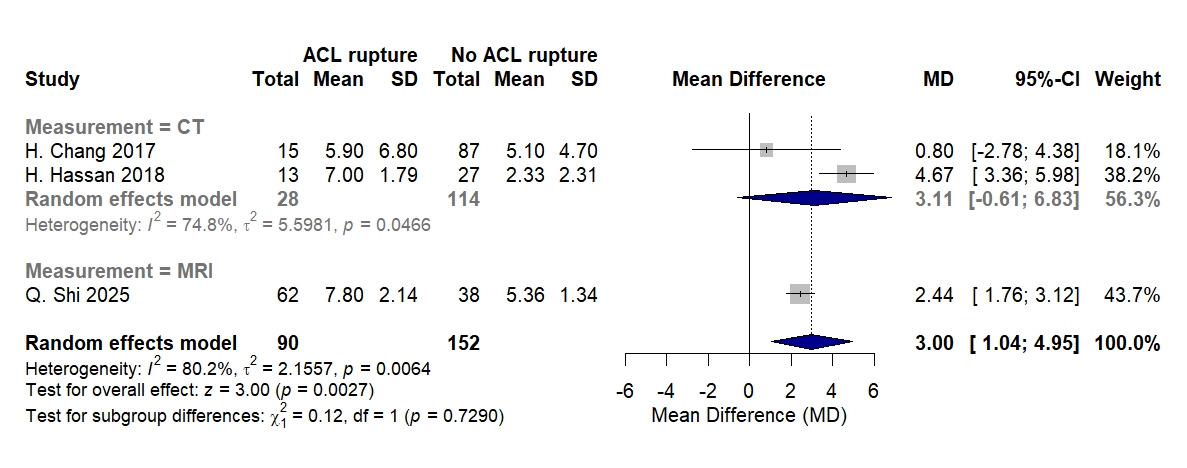


**B**

**Figure S13.** Subgroup analysis of the meta-analysis assessing mean difference (MD) in lateral plateau widening (LPW) between groups with and without anterior cruciate ligament (ACL) injury based on (A) reference standard and (B) measurement modality


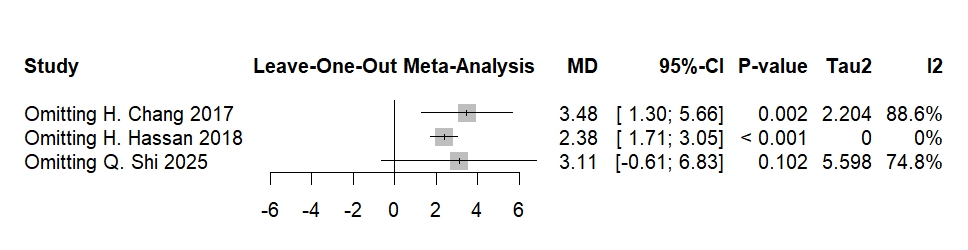


**Figure S14.** Leave-one-out sensitivity analyses of the meta-analysis assessing mean difference (MD) in lateral plateau widening (LPW) between groups with and without anterior cruciate ligament (ACL) injury


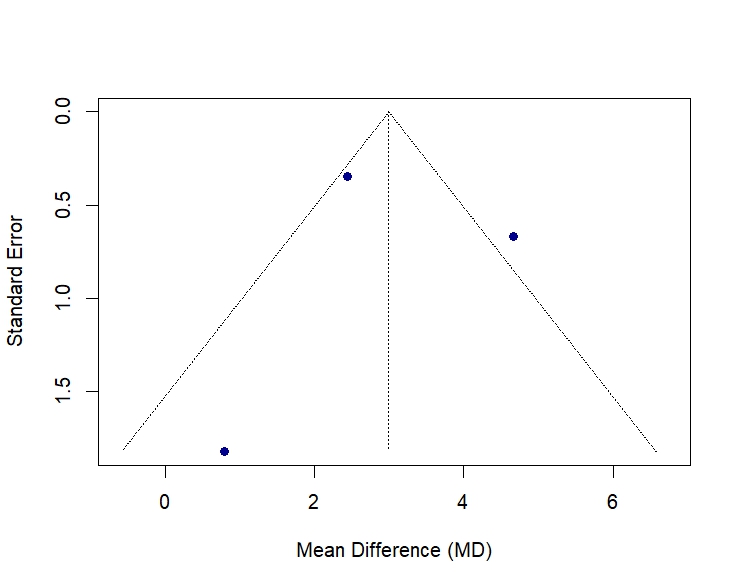


**Figure S15.** Funnel plot of mean difference (MD) in lateral plateau widening (LPW) between groups with and without anterior cruciate ligament (ACL) injury
